# Serum neurofilament measurement improves clinical risk scores for outcome prediction after cardiac arrest: results of a prospective study

**DOI:** 10.1186/s13054-021-03459-y

**Published:** 2021-01-20

**Authors:** Sabina Hunziker, Adrian Quinto, Maja Ramin-Wright, Christoph Becker, Katharina Beck, Alessia Vincent, Kai Tisljar, Giulio Disanto, Pascal Benkert, David Leppert, Hans Pargger, Stephan Marsch, Raoul Sutter, Nils Peters, Jens Kuhle

**Affiliations:** 1 Intensive Care Unit, University Hospital Basel, University of Basel, Petersgraben 4, 4031 Basel, Switzerland; 2 Medical Communication and Psychosomatic Medicine, University Hospital Basel, Klingelbergstrasse 23, 4031 Basel, Switzerland; 3 Division of Clinical Neurophysiology, Department of Neurology, University Hospital Basel, Petersgraben 4, 4031 Basel, Switzerland; 4 Medical Faculty, University of Basel, Klingelbergstrasse 61, 4056 Basel, Switzerland; 5 Neurologic Clinic and Policlinic, MS Center and Research Center for Clinical Neuroimmunology and Neuroscience Basel (RC2NB), University Hospital Basel, University of Basel, Basel, Switzerland; 6 Clinical Trial Unit Basel, Department of Clinical Research, University Hospital Basel, University of Basel, Basel, Switzerland

**Keywords:** Serum neurofilament, Cardiac arrest, Prognosis, CAHP, OHCA, Cardiopulmonary resuscitation

## Abstract

**Background:**

A recent study found serum neurofilament light chain (NfL) levels to be strongly associated with poor neurological outcome in patients after cardiac arrest. Our aim was to confirm these findings in an independent validation study and to investigate whether NfL improves the prognostic value of two cardiac arrest-specific risk scores.

**Methods:**

This prospective, single-center study included 164 consecutive adult after out-of-hospital cardiac arrest (OHCA) patients upon intensive care unit admission. We calculated two clinical risk scores (OHCA, CAHP) and measured NfL on admission within the first 24 h using the single molecule array NF-light^®^ assay. The primary endpoint was neurological outcome at hospital discharge assessed with the cerebral performance category (CPC) score.

**Results:**

Poor neurological outcome (CPC > 3) was found in 60% (98/164) of patients, with 55% (91/164) dying within 30 days of hospitalization. Compared to patients with favorable outcome, NfL was 14-times higher in patients with poor neurological outcome (685 ± 1787 vs. 49 ± 111 pg/mL), with an adjusted odds ratio of 3.4 (95% CI 2.1 to 5.6, *p* < 0.001) and an area under the curve (AUC) of 0.82. Adding NfL to the clinical risk scores significantly improved discrimination of both the OHCA score (from AUC 0.82 to 0.89, *p* < 0.001) and CAHP score (from AUC 0.89 to 0.92, *p* < 0.05). Adding NfL to both scores also resulted in significant improvement in reclassification statistics with a Net Reclassification Index (NRI) of 0.58 (*p* < 0.001) for OHCA and 0.83 (*p* < 0.001) for CAHP.

**Conclusions:**

Admission NfL was a strong outcome predictor and significantly improved two clinical risk scores regarding prognostication of neurological outcome in patients after cardiac arrest. When confirmed in future outcome studies, admission NfL should be considered as a standard laboratory measures in the evaluation of OHCA patients.

## Introduction

Despite the increased survival rates thanks to medical treatments, the mortality and risk for neurological deficits remains high for cardiac arrest patients [1–3]. Prognostication of outcome upon admission is difficult in these patients, yet, early identification of predictors for a poor outcome after out-of-hospital cardiac arrest (OHCA) would facilitate the therapeutic management, decision-making, and communication with relatives [4]. The associated high economic burden of comatose critically ill patients further increases the impact not just on an individual level, but also on society at large. For risk prediction of adverse clinical outcome, several clinical tools have been developed. Two scores were specifically developed for cardiac arrest patients including the Out-of-Hospital Cardiac Arrest score (OHCA) and the Cardiac Arrest Hospital Prognosis score (CAHP) [5–7]. These scores, however, still have limited accuracy and, thus, bear the risk of misclassifying patients. The improvement of these clinical scores by the addition of biomarkers reflecting pathophysiologic mechanisms of neural damage is an unmet medical need. Herein, different biomarkers indicating brain injuries after OHCA have been studied [8, 9], yet neuron-specific enolase (NSE) is currently the only biomarker recommended by guidelines as a prognostic blood marker for patients after cardiac arrest [9–12].

Recently, elevated levels of neurofilament light chain (NfL) in blood samples have been established as marker of neuronal damage in traumatic brain injury and many acute and chronic neurologic diseases [13–19]. NfL thus has potential to further improve assessment of neurological damage in OHCA patients. To date, one pilot study investigated this biomarker in cerebrospinal fluid [20] and a few studies in the blood of patients after cardiac arrest [21–24]. Two studies analyzed neurofilament levels with standard immunoassays and reported associations of NfL with poor neurological outcome in patients after cardiac arrest [21, 22]. One of these studies used an ultrasensitive single molecule array (Simoa) assay to test prognostication of neurological outcome after OHCA in a large cohort of patients [23]. Serum NfL levels at 24 h after cardiac arrest showed high discrimination regarding long-term poor neurological outcome with an area under the curve (AUC) of 0.94–0.95, which was better compared to other biomarkers (i.e., tau, neuron-specific enolase (NSE), and S100). Before wide-spread implementation of NfL, independent validation in a larger sample is mandatory.

Herein, the aim of this study was to externally validate the prognostic accuracy of NfL in a well characterized sample of patients after OHCA and to study whether NfL improves current OHCA specific risk scores, namely, the OHCA and CHAP scores.

## Methods

### Study setting

This is a prospective observational study including consecutive patients from November 2012 until February 2016 with data obtained in the COMMUNICATE trial at the University Hospital Basel, Switzerland. The main purpose of this study is to investigate novel biomarkers for risk stratification of OHCA patients. The methods used for this study have been published previously [7, 25, 26]. The study was approved by the local ethics committee. Patients or their relatives provided informed consent for study participation.

This manuscript adheres to the STROBE guidelines.

### Study population

We included consecutive patients after cardiac arrest who were admitted to the intensive care unit (ICU) of the University Hospital Basel, a Swiss tertiary academic medical center, into the study. We did not use any exclusion criteria regarding the patients’ characteristics and type or duration of the cardiac arrest except for patients having to be adults.

The treatment of patients regarding the cardiac arrest was based on the clinical routine in our ICU without interaction with the research team. Withdrawal of life-sustaining therapy was done per clinical routine after in-depth discussion within the treating team and the patients` family based on presumed patients`will, the medical and social situation and the patients prognosis as assessed by all available clinical, neurological and laboratory data (not including levels of NfL).

### Data collection

We calculated all scores as suggested [5, 6, 27, 28] and as described in more detail in a previous publication [7]. In brief, we used data collected on the first day of admission. Resuscitation data were collected from clinical records, including no-flow time [time from cardiac arrest to start of basic life support (BLS)], low-flow time [time from start of BLS to return of spontaneous circulation (ROSC)], initial rhythm, setting and location of arrest, epinephrine dose given, as well as information on whether bystanders observed the cardiac arrest and started cardiopulmonary resuscitation (CPR). Clinical parameters [e.g., Glasgow Coma Scale (GCS)], socio-demographics (e.g., age, gender) and comorbidities (i.e., coronary disease, congestive heart failure, hypertension, chronic obstructive pulmonary disease (COPD), malignant disease, diabetes, chronic kidney disease, liver failure) were recorded from medical records or during an interview with patients’ relatives. We also collected initial levels of routine blood markers, (e.g. pH, lactate, creatinine).

We also calculated two risk scores that were specifically developed for outcome prediction in the cardiac arrest patient population including the out-of-hospital cardiac arrest (OHCA) score and the Cardiac Arrest Hospital Prognosis (CAHP) score [5, 6]. The OHCA score was developed in 2006 in France and later validated in the US and Switzerland [5, 7, 29]. This score, which incorporates initial rhythm, no-flow and low-flow intervals, and admission levels of serum creatinine and lactate into a risk model, showed a high performance for outcome prediction. The CAHP score was developed later and includes additional resuscitation specific variables, such as location of cardiac arrest, epinephrine dose as well as initial blood ph values and age [30].

### Measurement of fluid biomarkers

We measured NfL in admission blood samples. After blood draw, all samples were pre-analytically processed, aliquoted, and frozen at − 80 °C. Serum NfL levels were measured in duplicates (intra-assay coefficients of variation < 20%) by Simoa NfL assay as described previously [31]. Inter-assay CVs were 11.8% and 14.1% for two native serum samples (104 pg/ml and 31 pg/ml), respectively. We also measured NSE levels using an Electro-Chemi-Luminescent-Immuno-Assay (ECLIA) kit (Roche Diagnostics, Rotkreuz, Switzerland).

### Outcome

Similar to previous studies, the primary endpoint was neurological outcome at hospital discharge assessed with the cerebral performance category (CPC) Score [32]. In line with previous studies, levels 1 (good recovery) and 2 (moderate disability) were considered as favourable neurological outcome, whereas levels 3 (severe disability), 4 (vegetative state) and 5 (death) were defined as unfavourable outcome [5, 6]. We also measured the modified Rankin Scale (mRS) at discharge, which was used as secondary endpoint. We defined levels 0 (no symptoms), 1 (no significant disability despite symptoms; able to carry out all usual duties and activities), 2 (slight disability; unable to carry out all previous activities, but able to look after own affairs without assistance), and 3 (moderate disability; requiring some help, but able to walk without assistance) as favourable outcome, and levels 4 (moderately severe disability, unable to walk and attend to bodily needs without assistance), 5 (severe disability; bedridden, incontinent and requiring constant nursing care and attention) and 6 (dead) as unfavourable outcome [33, 34]. A further secondary endpoint was all-cause mortality at 30 days after discharge.

### Statistical analysis

To characterise the patient cohort, descriptive statistics including means (± SD), medians and inter-quartile ranges were used for continuous variables as appropriate, whereas frequencies were reported for binary or categorical variables. We calculated univariable and multivariable logistic regression models to evaluate the association of NfL levels with the primary and secondary endpoints. Data were inspected for normality by use of Q–Q plots. To achieve a normal distribution, data of NfL levels were log transformed with a base of 10. Odds ratios (OR) and 95% confidence intervals (CI) were reported as a measure of association. For regression analysis, we also used quartiles of NfL to study association with outcome. Covariates used in the multivariate analysis were selected based on prior evidence of an association with unfavourable neurological outcome for patients with OHCA. Multivariate models were adjusted for age, gender and comorbidities. In a further step, we additionally included OHCA and CAHP scores (which are based on several prognostic parameters) into the models. In addition to regression analysis to study strength of association, we also calculated area under the ROC curve (AUC) to provide a measure of discrimination for all parameters. To understand whether NfL would improve established risk scores, we also calculated AUCs of combined models (score plus NfL) to see whether NfL would improve discrimination of the scores. We also calculated the Net Reclassification Index (NRI) across risk categories of 5%, 10%, 30%, 50% and 80% and the Integrated Discrimination Index (IDI) as proposed by Pencina and colleagues [35].

We also investigated the prognostic performance of NfL regarding sensitivity, specificity, positive and negative predictive values and likelihood ratios at three cut-offs (25, 50, 75 pg/ml), which were close to the median NfL as well as the lower and upper interquartile range. Additionally, we investigated two cut-offs to maximize specificity and sensitivity.

STATA 15.0 was used for all statistical analyses and a two-sided *p *value of < 0.05 was considered significant.

## Results

### Baseline characteristics

From the 164 included patients, 98 (60%) had poor neurological outcome and 91 (55%) patients died. The baseline characteristics of the cohort overall and stratified based on neurological outcome are shown in Table 1. Overall, the mean age was 63 years and 28.7% were female. Patients showed relevant comorbidities and cardiovascular risk factors. Patients with poor neurological outcome had less pre-existing coronary heart disease and a higher incidence of diabetes mellitus and malignant disease than patients with good neurological outcome. In patients with poor neurological outcome the cardiac arrest was more often unwitnessed, and they less frequently received bystander CPR and further had longer no-flow and low flow time than patients with a good neurological outcome. In terms of the initial clinical parameter, patients with poor neurological outcome had a lower GCS, lower pH levels and higher lactate levels than patients with a good neurological outcome.

**Table 1:** Baseline characteristics

Factor	CPC All	Good neurological outcome, CPC 1–2	Poor neurological outcome, CPC 3–5	*p *value
*N*, *n* (%)	164 (100%)	66 (40%)	98 (60%)	
*Sociodemographics*				
Age, mean (SD)	63 (15)	60 (17)	65 (14)	0.027
Male, *n* (%)	117 (71.3%)	55 (83%)	62 (63%)	0.008
*Comorbidities*				
Coronary heart disease, *n* (%)	109 (66.5%)	50 (76%)	59 (60%)	0.044
Congestive heart failure, *n* (%)	20 (12.2%)	7 (11%)	13 (13%)	0.81
COPD, *n* (%)	15 (9.1%)	3 (5%)	12 (12%)	0.11
Liver disease, *n* (%)	3 (1.8%)	0 (0%)	3 (3%)	0.27
Hypertension, *n* (%)	86 (52.4%)	37 (56%)	49 (50%)	0.52
Diabetes, *n* (%)	39 (23.8%)	9 (14%)	30 (31%)	0.015
Chronic kidney disease, *n* (%)	24 (14.6%)	8 (12%)	16 (16%)	0.51
Malignant disease, *n* (%)	14 (8.5%)	2 (3%)	12 (12%)	0.047
Neurological disease, *n* (%)	13 (7.9%)	2 (3%)	11 (11%)	0.077
*Resuscitation measures*				
No-flow time, min, mean (SD)	4.22 (5.93)	1.70 (2.96)	6.02 (6.83)	< 0.001
Low-flow time, min, mean (SD)	20.42 (15.13)	16.53 (14.30)	23.12 (15.17)	0.006
*Cardiac arrest setting*				
At home	63 (38.4%)	16 (24%)	47 (48%)	< 0.001
In public	78 (47.6%)	43 (65%)	35 (36%)	
In hospital	23 (14.0%)	7 (11%)	16 (16%)	
Witnessed	137 (83.5%)	63 (95%)	74 (76%)	< 0.001
Bystander CPR	103 (62.8%)	51 (77%)	52 (53%)	0.002
*Initial heart rhythm*				
Ventricular tachycardia	6 (3.7%)	2 (3%)	4 (4%)	< 0.001
Ventricular fibrillation	88 (53.7%)	53 (80%)	35 (36%)	
Asystole	35 (21.3%)	2 (3%)	33 (34%)	
Pulseless electrical activity	29 (17.7%)	6 (9%)	23 (23%)	
Unknown	6 (3.7%)	3 (5%)	3 (3%)	
Epinephrine during resuscitation (mg), mean (SD)	2.18 (2.55)	1.08 (1.76)	2.96 (2.73)	< 0.001
Targeted temperature management (TTM), *n* (%)	109 (66.5%)	40 (61%)	69 (70%)	0.19
*Initial clinical parameter and levels of routine blood markers*				
GCS, mean (SD)	7.3 (4.1)	7 (5)	4 (1)	< 0.001
pH, mean (SD)	7.25 (0.12)	7.29 (0.09)	7.22 (0.13)	0.003
Lactate, mean (SD)	7.3 (4.1)	5.9 (3.3)	8.3 (4.2)	< 0.001
NfL (pg/ml), mean (SD)	429 (1415)	49 (111)	685 (1787)	0.004
NfL (pg/ml), median (IQR)	51 (21, 173)	27 (13, 46)	116 (41, 330)	< 0.001

Data presented as *n* (%) or mean (standard deviation) and median (Inter Quartile Range, IQR). COPD, chronic obstructive pulmonary disease; CPR, cardiopulmonary resuscitation; NfL, Neurofilament Light Chain; GCS, Glasgow Coma Scale; Neurological disease includes cerebrovascular diseases (e.g., stroke, brain haemorrhage), degenerative diseases such as multiple sclerosis or Parkinson’s disease and peripheral neurological disease

### Primary endpoint: Poor neurological outcome measured with the cerebral performance category (CPC) score

NfL levels were higher in patients with poor neurological outcome compared to patients with good neurological outcome [mean/median 685/116 (SD ± 1787) vs mean/median 49/27 (SD ± 111), *p* = 0.004] with an univariate OR (of log-transformed NfL) of 2.9 (95% CI 2.0 to 4.3, *p* < 0.001) and a multivariate OR of 3.4 (95% CI 2.1 to 5.6, *p* < 0.001) after adjusting for age, gender and comorbidities (Table 2). Further, the risk for poor neurological outcome increased more than 40-fold for patients in the highest quartile compared to the lowest quartile of NfL concentrations [OR 47.1 (95% CI 9.8 to 227.0), *p* < 0.001].

**Table 2:** Comparison between scores to predict primary and secondary endpoints

	Good neurological outcome (CPC 1–2)	Poor neurological outcome (CPC 3–5)	*p *value	Univariate		Multivariate^a^ adjusted	NFL and OHCA score: multivariate^a^ adjusted		NFL and CAHP score: multivariate^a^ adjusted	
	*n* = 66	*n* = 98		OR (95% CI), *p*	AUC	OR (95% CI), *p*	OR (95% CI), *p*	AUC combined with NFL	OR (95% CI), *p*	AUC combined with NFL
*Primary endpoint: neurological outcome (CPC)*										
NfL, mean (SD)	49 (111)	685 (1787)	0.004	2.93 (2.01, 4.27), *p* < 0.001	0.82	3.44 (2.13, 5.55), *p* < 0.001	2.81 (1.69, 4.67), *p* < 0.001	0.89	3.45 (1.89, 6.29), *p* < 0.001	0.92
NfL, median (IQR)	27 (13, 46)	116 (41, 330)	< 0.001							
*Quartiles of NFL*										
1st quartile	29 (44%)	12 (12%)	< 0.001	*Reference group*		*Reference group*	*Reference group*		*Reference group*	
2nd quartile	25 (38%)	16 (16%)		1.55 (0.62, 3.88), *p* = 0.35		1.35 (0.46, 4.03), *p* = 0.59	1.02 (0.28, 3.7), *p* = 0.97		1.01 (0.24, 4.23), *p* = 0.98	
3rd quartile	10 (15%)	31 (32%)		7.49 (2.81, 19.96), *p* < 0.001		5.59 (1.65, 18.94), *p* = 0.006	3.33 (0.82, 13.41), *p* = 0.091		3.99 (0.84, 18.96), *p* = 0.08	
4th quartile	2 (3%)	39 (40%)		47.12 (9.78, 227.01), *p* < 0.001		127.26 (16.53, 979.47), *p* < 0.001	92.39 (8.47, 1007.63), *p* < 0.001		140.48 (10.5, 1880.26), *p* < 0.001	
OHCA, mean (SD)	11 (16)	32 (17)	< 0.001	1.08 (1.05, 1.1), *p* < 0.001	0.82	1.08 (1.05, 1.11), *p* < 0.001	1.07 (1.04, 1.11), *p* < 0.001			
CAHP, mean (SD)	118 (41)	182 (38)	< 0.001	1.04 (1.03, 1.05), *p* < 0.001	0.89	1.04 (1.03, 1.05), *p* < 0.001			1.04 (1.02, 1.06),*p* < 0.001	

	Good neurological outcome (mRs 0–2)	Poor neurological outcome (mRs 3–6)	*p *value	Univariate		Multivariate^a^ adjusted	NFL and OHCA score: multivariate^a^ adjusted		NFL and CAHP score: multivariate^a^ adjusted	
	n = 53	n = 111		OR (95% CI), *p*	AUC	OR (95% CI), *p*	OR (95% CI), *p*	AUC combined with NFL	OR (95% CI), *p*	AUC combined with NFL
*Secondary endpoint: Neurological outcome (mRs)*										
NFL, mean (SD)	51 (123)	609 (1691)	0.018	2.72 (1.85, 3.98), *p* < 0.001	0.8	3.01 (1.88, 4.83), *p* < 0.001	2.47 (1.52, 4.04), *p* < 0.001	0.87	2.78 (1.64, 4.72), *p* < 0.001	0.88
NfL, median (IQR)	21 (12, 40)	92 (37, 284)	< 0.001							
*Quartiles of NFL*										
1st quartile	26 (49.1%)	15 (13.5%)	< 0.001	*Reference group*		*Reference group*	*Reference group*		*Reference group*	
2nd quartile	18 (34.0%)	23 (20.7%)		2.21 (0.91, 5.37), *p* = 0.078		2.11 (0.73, 6.1), *p* = 0.17	1.8 (0.56, 5.83), *p* = 0.326		1.89 (0.55, 6.46), *p* = 0.309	
3rd quartile	7 (13.2%)	34 (30.6%)		8.42 (3, 23.64), *p* < 0.001		6.14 (1.73, 21.8), *p* = 0.005	3.6 (0.9, 14.41), *p* = 0.07		3.97 (0.94, 16.7), *p* = 0.06	
4th quartile	2 (3.8%)	39 (35.1%)		33.8 (7.13, 160.31), *p* < 0.001		66.08 (9.66, 452.17), *p* < 0.001	42.17 (4.72, 376.98), *p* = 0.001		53.32 (5.52, 514.78), *p* = 0.001	
OHCA, mean (SD)	11 (16)	30 (18)	< 0.001	1.06 (1.04, 1.09), *p* < 0.001	0.79	1.07 (1.04, 1.1), *p* < 0.001	1.06 (1.03, 1.08), *p* < 0.001			
CAHP, mean (SD)	117 (42)	175 (42)	< 0.001	1.03 (1.02, 1.04), *p* < 0.001	0.84	1.03 (1.02, 1.04), *p* < 0.001			1.03 (1.02, 1.04), *p* < 0.001	

	Survivors	Non-Survivors	*p *value	Univariate		Age, gender, comorbidities adjusted	NFL and OHCA score: multivariate^a^ adjusted		NFL and CAHP score: multivariate^a^ adjusted	
	*n* = 73	*n* = 91		OR (95% CI), *p*	AUC	OR (95% CI), *p*	OR (95% CI), *p*	AUC combined with NFL	OR (95% CI), *p*	AUC combined with NFL
*Secondary endpoint: 30-day mortality*										
NfL, mean (SD)	136 (701)	664 (1763)	0.017	2.77 (1.94, 3.95), *p* < 0.001	0.83	2.97 (1.91, 4.62), *p* < 0.001	2.42 (1.54, 3.8), *p* < 0.001	0.87	2.59 (1.6, 4.2), *p* < 0.001	0.89
NfL, median (IQR)	27 (13, 47)	128 (45, 330)	< 0.001							
*Quartiles of NFL*										
1st quartile	33 (45%)	8 (9%)	< 0.001	*Reference group*		*Reference group*	*Reference group*		*Reference group*	
2nd quartile	25 (34%)	16 (18%)		2.64 (0.98, 7.14), *p* = 0.056		2.47 (0.75, 8.1), *p* = 0.135	2.29 (0.62, 8.42), *p* = 0.211		2.32 (0.58, 9.3), *p* = 0.235	
3rd quartile	11 (15%)	30 (33%)		11.25 (3.99, 31.71), *p* < 0.001		7.85 (2.19, 28.09), *p* = 0.002	5.49 (1.39, 21.76), *p* = 0.015		5.87 (1.35, 25.57), *p* = 0.019	
4th quartile	4 (5%)	37 (41%)		38.16 (10.52, 138.44), *p* < 0.001		92.29 (15.16, 561.71), *p* < 0.001	49.33 (7.29, 333.79), *p* < 0.001		65.12 (8.32, 509.95), *p* < 0.001	
OHCA, mean (SD)	14 (18)	32 (17)	< 0.001	1.06 (1.04, 1.09), *p* < 0.001	0.77	1.07 (1.04, 1.1), *p* < 0.001	1.05 (1.03, 1.08), *p* < 0.001			
CAHP, mean (SD)	123 (43)	182 (38)	< 0.001	1.03 (1.02, 1.04), *p* < 0.001	0.85	1.03 (1.02, 1.04), *p* < 0.001			1.03 (1.02, 1.04), *p* < 0.001	

Data presented as mean (SD) and median (Inter Quartile Range, IQR).; NfL = Neurofilament Light Chain (pg/ml); AUC = area under the curve; CAHP = Cardiac Arrest Hospital prognosis (-score); CPC = cerebral performance category; OHCA = Out-of-Hospital Cardiac Arrest (-score); OR = odds ratio; ROC = receiver operating characteristics curve

^a^Adjusted for age, gender, comorbidities

NfL showed a comparable prognostic discrimination with an AUC of 0.82 to both established clinical risk scores. Adding NfL to the risk scores further improved discrimination between good and poor neurological outcome (from AUC 0.82 with OHCA score alone to 0.89 in combination with NfL, *p* < 0.001, from AUC 0.89 with CAHP score alone to 0.92 in combination, *p* < 0.05).

NfL also showed a significant improvement in regard to the NRI of 0.58 (*p* < 0.001) for OHCA (among patients with poor outcome, adding NfL increased the risk in the statistical model in 30%, while decreasing the risk in 11%; and among patients with favorable outcome, adding NfL decreased the risk of the model in 54% while increasing it in 15%). For the CAHP score, there was also a strong improvement with an NRI of 0.83 (*p* < 0.001) (among patients with poor outcome, adding NfL increased the risk in the statistical model in 36%, while decreasing the risk in 7%; and among patients with favorable outcome, adding NfL decreased the risk of the model in 67% while increasing it in 12%). The IDI for OHCA were 0.15 (*p* < 0.001) and for CAHP 0.25 (*p* < 0.001).

Table 3 shows sensitivity and specificity, positive and negative predictive value as well as positive and negative likelihood ratios for NfL at three different cut-offs. At the calculated optimal cut-off (Youden-Index) of 50 pg/ml, we found a corresponding sensitivity of 72.4% and specificity of 81.8%, with 85.5% positive predictive value and 66.7% negative predictive value for poor neurological outcome. Further, we calculated prognostic measures based on receiver operating characteristic (ROC) curve analysis for the cut-offs at a NfL level of 25 pg/ml and 75 pg/ml. For the cut-off of 25 pg/ml, the sensitivity was 87.8% and specificity 47%, with 71.1% positive predictive value and 72.1% negative predictive value. Finally, a cut-off of 75 pg/ml revealed a sensitivity of 61.2%, specificity of 90.9%, a 90.9% positive predictive value, and a negative predictive value of 61.2%.

**Table 3:** Performance of NfL at different cut-off points to predict neurological Outcome

	CPC score			mRS			Mortality 30 days		
	NfL cut-off 25 pg/ml	NfL cut-off 50 pg/ml	NfL cut-off 75 pg/ml	NfL cut-off 25 pg/ml	NfL cut-off 50 pg/ml	NfL cut-off 75 pg/ml	NfL cut-off 25 pg/ml	NfL cut-off 50 pg/ml	NfL cut-off 75 pg/ml
Poor outcome/total per group	12/43	15/38	71/83	16/43	21/38	74/83	8/43	15/38	68/83
Sensitivity Pr(+ A)	87.80%	72.40%	61.20%	85.60%	66.70%	55.00%	91.20%	74.70%	63.70%
Specificity Pr(− N)	47.00%	81.80%	90.90%	50.90%	83.00%	90.60%	47.90%	79.50%	89.00%
ROC area (sens. + spec.)/2	0.67	0.77	0.76	0.68	0.75	0.73	0.7	0.77	0.76
Likelihood ratio ( +) Pr(+ A)/Pr(+ N)	1.65	3.98	6.73	1.74	3.93	5.83	1.75	3.64	5.82
Likelihood ratio (−) Pr(− A)/Pr(− N)	0.26	0.34	0.43	0.28	0.4	0.5	0.18	0.32	0.41
Odds ratio LR( +)/LR(−)	6.35	11.83	15.79	6.17	9.78	11.71	9.56	11.43	14.28
Positive predictive value Pr(A +)	71.10%	85.50%	90.90%	78.50%	89.20%	92.40%	68.60%	81.90%	87.90%
Negative predictive value Pr(N −)	72.10%	66.70%	61.20%	62.80%	54.30%	49.00%	81.40%	71.60%	66.30%

Data presented as mean (SD); NfL = Neurofilament Light Chain (pg/ml); CAHP = Cardiac Arrest Hospital prognosis (-score); CPC = cerebral performance category; mRS = modified Rankin Scale; LLR +  = positive likelihood ratio; LLR- = negative likelihood ratio; NPV = negative predictive value; PPV = positive predictive value

### Secondary endpoints

Secondary endpoints were the modified Rankin Scale (mRS) and all-cause mortality at hospital discharge (Table 2).

In terms of the mRS, the mean/median NfL levels were also significantly higher in patients with poor neurological outcome [609 pg/ml/92 (SD ± 1691) vs 51/21 pg/ml (SD ± 123), *p* = 0.018] with a corresponding univariate OR of 2.72 (95% CI 1.85 to 3.98). These results stayed robust after adjusting in the full model [OR of 3.01 (95% CI 1.88 to 4.83)].

NfL showed a good prognostic discrimination for poor neurological outcome assessed with the mRS with an AUC of 0.80 which further improved in combination with the two risk scores to an AUC of 0.87 with OHCA and to an AUC of 0.88 with CAHP.

In Table 3 at the calculated optimal cut-off of 50 pg/ml, sensitivity was 66.7% and specificity was 83%, with 89.2% positive predictive value and 54.3% negative predictive value for poor neurological outcome. Further, we calculated the prognostic measures based on ROC curve analysis for the cut-offs at a NfL level of 25 pg/ml and 75 pg/ml. For the cut-off of 25 pg/ml, the calculated sensitivity was 85.6% and the specificity 50.9%, with 78.5% positive predictive value and 62.8% negative predictive value. Further, a cut-off of 75 pg/ml showed a sensitivity of 55%, specificity 90.6%, a 92.4% positive predictive value and a negative predictive value of 49%.

Similarly, NfL was significantly associated with mortality, also after adjusting in the full model [mean/median 664/128 (SD 1763) vs mean/median 136/27 (SD 701), *p* = 0.017] with a good prognostic discrimination (AUC 0.83). Also, we found an improvement of the prognostic value by combination of NfL with the OHCA and the CAHP risk scores (AUC from 0.77 to 0.87 and AUC 0.85 to 0.89 respectively).

At the calculated optimal cut-off of 50 pg/ml, we found a sensitivity of 74.7% and specificity of 79.5%, with 81.9% positive predictive value and 71.6% negative predictive value for poor neurological outcome. For the cut-off of 25 pg/ml, the sensitivity was 91.2% and specificity 47.9%, with 68.6% positive predictive value and 81.4% negative predictive value. Finally, a cut-off of 75 pg/ml revealed a sensitivity of 63.7%, specificity of 89%, an 87.9% positive predictive value, and a negative predictive value of 66.3%. Specificity was further increased to 98.5% at NfL cut-off of 229 pg/ml (corresponding sensitivity 37%). Likewise, sensitivity was 98.0% at NfL cut-off of 13 pg/ml (corresponding specificity 23%).

**Fig. 1:**
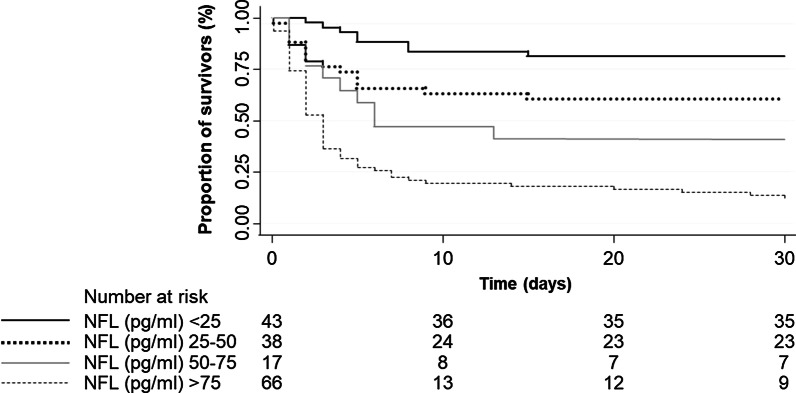
Kaplan Meier curves for 30d all-cause mortality based on the three different NfL cut-offs. Data is presented as number (n) percentage (%); NfL: Neurofilament Light Chain

Figure 1 displays Kaplan–Meier curves for 30-day all-cause mortality based on the three different NfL cut-offs.

**Fig. 2:**
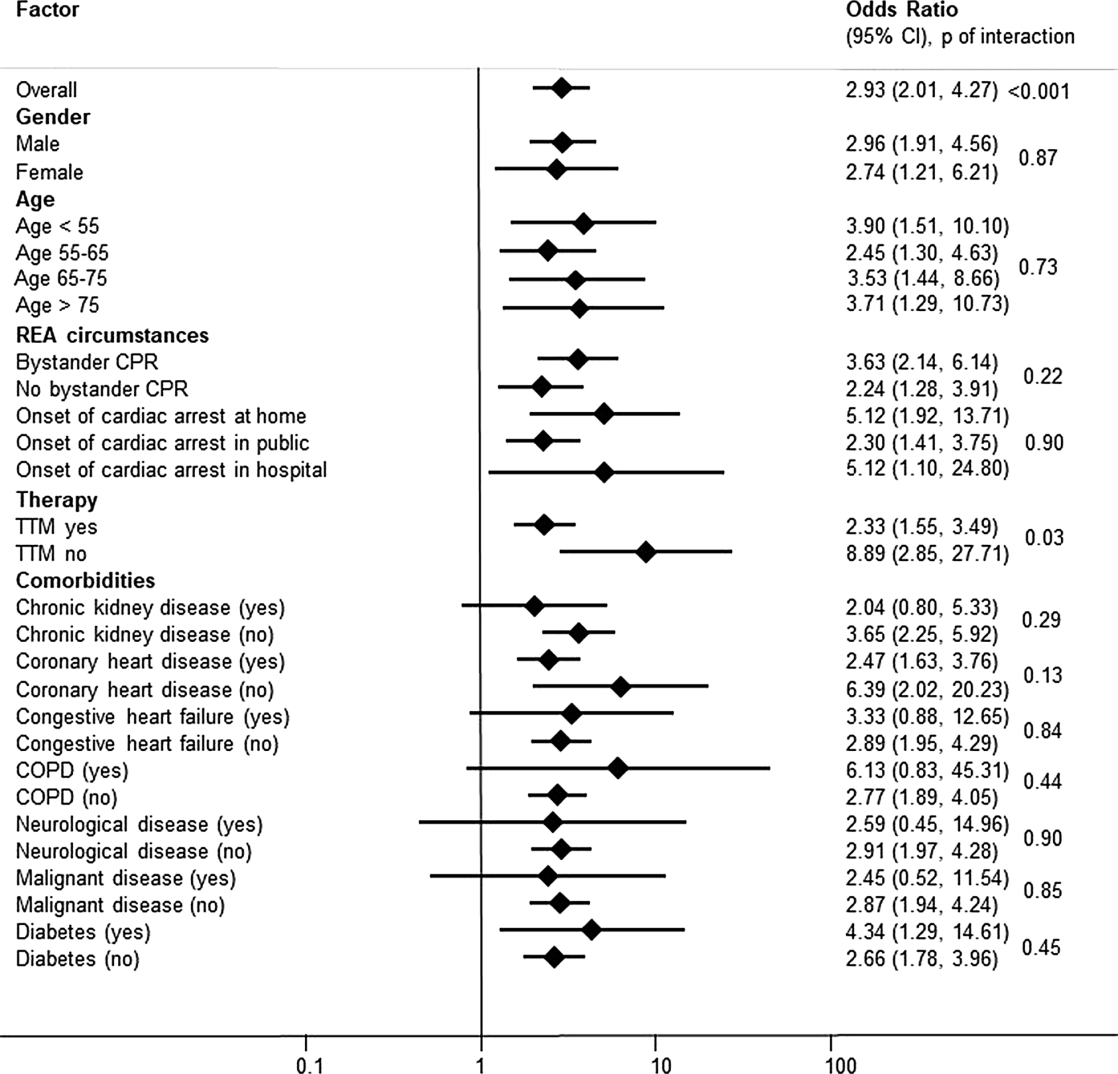
Subgroup analysis of NfL for primary endpoint (neurological outcome). Data is presented as univariable odds ratio (OR) and 95% confidence interval (95% CI). NfL: Neurofilament Light Chain; TTM: Targeted Temperature Management, COPD: Chronic obstructive pulmonary disease

We additionally performed subgroup analyses to evaluate differences in associations of NfL with neurological outcome in specific patient groups. Results were also robust in these subgroups for different variables (Fig. 2).

**Fig. 3:**
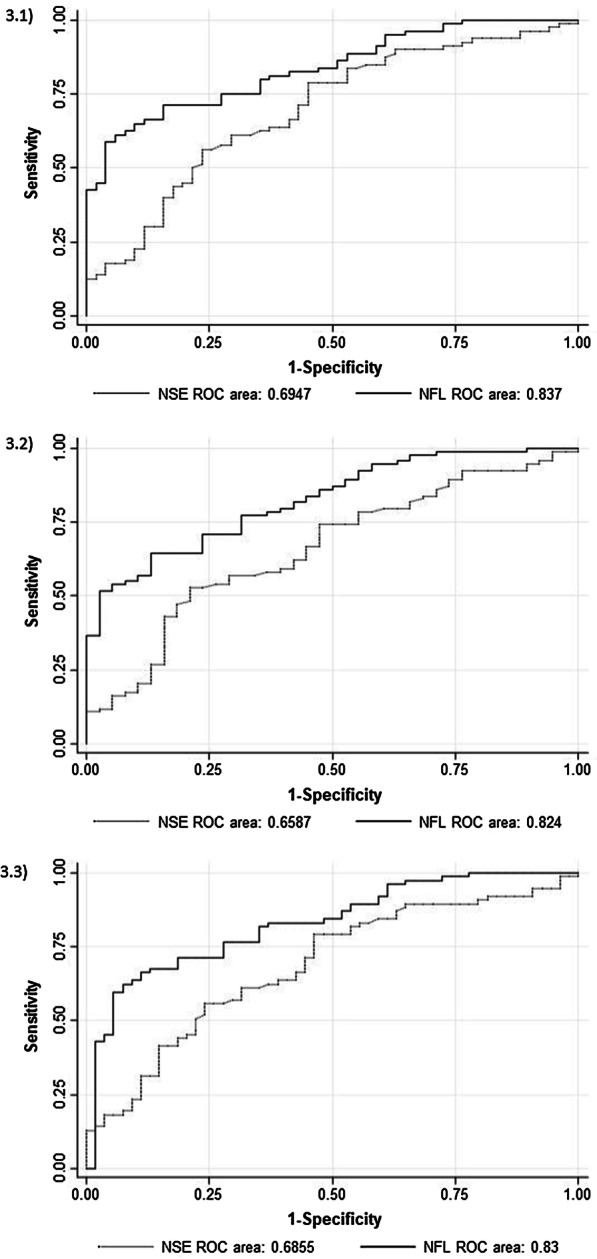
Areas under the curve (AUC) for combination of NfL with NSE in a subgroup of 131 patients. AUC for NSE compared with the AUC NfL for 3.1) the primary endpoint neurological outcome measured with cerebral performance category (CPC), 3.2) the endpoint neurological outcome measured with the modified ranking scale (mRS), 3.3) endpoint neurological mortality; ROC, receiver operating characteristic; NfL: Neurofilament Light Chain; NSE: Neuron specific enolase

We also compared NfL to other blood biomarkers regarding the prognostic accuracy regarding our primary endpoint. First, we compared NfL to initial lactate and found a significant higher AUC of NfL (AUC 0.82 vs 0.68, *p* = 0.02). NfL was also significantly better regarding neurological outcome measured with the mRS (AUC 0.80 vs 0.66, *p* = 0.02) and for mortality (AUC 0.83 vs 0.65, *p* = 0.002) Second, we compared NfL to NSE in patients who had available measurement of NSE on admission (n = 131). In this group, we found a significantly better prognostic performance of NfL compared to NSE (AUC NfL 0.84 vs. NSE 0.69, *p* = 0.01). Admission NfL also had a significantly higher AUC compared to admission NSE for neurological outcome measured with the mRS (AUC 0.82 vs 0.66, *p* = 0.008) and for mortality (AUC 0.83 vs 0.69, *p* = 0.014) (Fig. 3).

## Discussion

In this prospective cohort of OHCA patients, we externally validated previous research and found that serum NfL level is a reliable predictor for poor neurological outcome and in-hospital mortality. Associations of NfL and outcome also remained significant in models adjusted for age, gender, comorbidities and cardiac-arrest specific risk scores. In addition, our data show that NfL significantly improved the prognostic value of two established cardiac arrest specific scoring systems (OHCA and CHAP Score) to predict outcome after cardiac arrest. Results of the previous derivation studies and our prospective validation suggest that serum NfL has high potential to improve patient care regarding early risk stratification in the population of cardiac arrest patients.

Neurofilaments have emerged in recent years as biofluid markers of neuronal damage which causes the release of these intracellular cytoskeleton proteins into cerebrospinal fluid and blood. Due to the development of ultrasensitive methods such as Simoa, levels are now quantifiable in serum or plasma, allowing longitudinal measurements for monitoring purposes. NfL is currently the most widely used marker among the three sizes of neurofilaments, mainly due to superior assay performance. NfL levels closely reflect the rate and degree of disease acuity in many chronic and acute illnesses of the central nervous system, as well as in traumatic brain injury [13]. In the case of OHCA, NfL could be a synergistic prognostic indicator for poor outcome due to first an increase in its level in the initial period of cardiac-arrest induced anoxic brain injury, and later due to an increase caused by neurologically stunned myocardium which again causes brain injury [36]. In addition, lifestyle and cardiovascular health have been shown to influence NFL baseline levels and could thus help to identify a high-risk group of patients regarding cardiovascular outcome [37]. Further, in many of these conditions NfL levels are now established on the group level as predictors for later functional neurological and neuropsychological outcome. Importantly, despite these promising results in other neurological illnesses, there has been relatively little clinical data regarding NfL in OHCA patients.

Our results are in line with two previous studies indicating that NfL is a good novel marker for prognosticating neurological outcome after cardiac arrest [21, 23]. While these studies focused on long-term outcomes after six months, our data suggest that NfL also helps to predict short-term clinical outcomes and thus may help navigate the therapeutic management in an early stage after cardiac arrest. We also investigated the potential of NfL to improve the cardiac specific OHCA and the CAHP scores [5, 25, 29]. Importantly, our data show that NfL measured early in the course after cardiac arrest further improves the discrimination of these clinical risk scores. An important advantage of these scores is that their calculation is based on initial ICU parameters that are readily available upon patient admission, allowing to support clinical decision-making regarding the initial management of OHCA patients. At this time, prognostication is particularly challenging as patients are often unconscious, limiting the clinical assessment. Early prognostic information, however, may help to inform relatives about expected risks and thus influence discussions about potential withdrawal of therapy. Herein, providing prognostic information with a high specificity regarding death and/or poor neurological outcome is of high importance as such decisions have huge consequences. Combining prognostic information from both, clinical scores and specific biomarkers may further improve prognostication and risk stratification. Such a strategy may further improve the current recommendation of taking a multimodal approach and not prognosticating patients before 72–96 h. Clearly, interventional research is needed to understand how prognostic information influences these decisions in more detail.

In patients after cardiac arrest, NSE is currently the only biomarker recommended for prognostication by European and American guidelines [38–40]. In a previous study, we found that NSE is highly predictive for adverse outcome when measured at day 3 of admission, while initial NSE levels measured on day 1 provided only little prognostic information [41]. The current analysis as well as another recent study found that the prognostic value of NSE on admission was inferior to NfL indicating that NfL may be a more accurate outcome marker early in the course of disease [23]. This may be explained by differences in marker kinetics and other factors such as influence of hemolysis on measurement characteristics. Importantly, admission NfL had a better prognostic performance to predict outcome compared to NSE. Thus, future guidelines should consider including NfL for early prognostication in patients after cardiac arrest as NSE shows only suboptimal results in the early course of disease.

In our study, NfL was measured at baseline within 24 h after onset of cardiac arrest. Literature regarding the kinetics of serum NfL after cardiac arrest is still sparse, especially within the early course before 24 h or after 72 h. In previous stroke and cardiac arrest populations, NfL levels in patients with poor outcome were nearly doubled from 24 to 48 h after cardiac arrest, reaching a steady state between 48 and 72 h [19, 23]. As a limitation, we did not measure NfL at other time points than admission, but possibly looking at kinetics could further improve its prognostic value. Importantly, NfL levels may increase after different types of central nervous system and peripheral nervous system injuries and are thus not specific for cardiac arrest. We only assessed neurological comorbidities as an overall item without specifying these in more detail. However, when adjusting for neurological comorbidities, result stayed robust suggesting no confounding in this regard. Further we did not find an effect modification by neurological comorbidities.

## Strength and limitations

Strengths of this study are the representative sample size with prospective and consecutive inclusion of patients and blinded analysis of blood markers. There are, however, some limitations: First, this is an observational study and is thus only hypothesis generating. We also did not have data on more advanced neurological examinations (e.g., EEG, SSEP) and on cause of death (i.e., brain injury vs. other causes) which would have been interesting regarding NfL performance. Also, no information regarding falls and brain/head trauma was available, which could influence NfL results. Second, we did not measure NfL during follow-up and thus cannot make any statements about its kinetics. Third, the time interval between ROSC and collection of NfL measurements has not been recorded. This measurement would have been valuable to study the dynamics over the first 24 h after ICU admission of NfL levels in our patient group. Forth, we had an important overlap of patients with unfavorable outcome and non-survivors limiting the interpretation of results. Fifth, we did not differentiate the cause of death (i.e., withdrawal of therapy vs. re-arrest or complications). Still, usually, withdrawal of therapy would be expected to occur later in course after rewarming of the patient and discussion with the family, and not within the first 24 h. Also, while prognostic information regarding routine parameters were available to physicians and may have influenced withdrawal decisions, biomarker levels of NfL as well as OHCA and CAHP scores are not part of our routine care and were thus not routinely available to the treating team and did thus not influence decisions. Finally, external validation of the NfL cutoff levels proposed by our analysis is necessary before wide-spread use in clinical practice.

## Conclusions

Admission NfL was a strong outcome predictor and significantly improved two clinical risk scores regarding prognostication of neurological outcome in patients after cardiac arrest. When confirmed in future outcome studies, admission NfL should be considered as a standard laboratory measures in the evaluation of OHCA patients.

## Data Availability

The datasets used and/or analyzed during the current study are available from the corresponding author on reasonable request.

## Abbreviations

AUC: Area under the curve
BLS: Basic life support
CAHP: Cardiac Arrest Hospital Prognosis
CI: Confidence interval
COPD: Chronic obstructive pulmonary disease
CPC: Cerebral performance category
CPR: Cardiopulmonary resuscitation
GCS: Glasgow Coma Scale
ICU: Intensive care
IQR: Inter Quartile Range
mRS: Modified Rankin Scale
NfL: Neurofilament light chain
NPV: Negative predictive value
NSE: Neuron-specific enolase
OHCA: Out-of-hospital cardiac arrest
OR: Odds ratio
OR: Odds ratio
PPV: Positive predictive value
ROC: Receiver-operating characteristic
ROSC: Return of spontaneous circulation
SD: Standard deviation
